# Preventing physician burnout: satisfaction or something more?

**DOI:** 10.1186/s13584-019-0303-y

**Published:** 2019-03-27

**Authors:** Stuart Slavin

**Affiliations:** Senior Scholar for Well-Being, Accreditation Council for Graduate Medical Education, 401 N. Michigan Ave., Suite 2000, Chicago, IL 60611 USA

**Keywords:** Burnout, Physicians, Pediatricians, Meaning

## Abstract

Physician burnout and depression have been recognized as serious international problems and the secondary costs of poor physician mental health are substantial. Interventions to address this problem can be split into two categories: those focusing on the individual, and those addressing the work environment. Individual-focused programs often include instruction in mindfulness, nutrition, and exercise, while those in the work environment have focused largely on stressors such as administrative burden, electronic health records, and productivity pressures.

The recent IJHPR article entitled “Burnout and intentions to quit the practice among community pediatricians: Associations with specific professional activities”, by Grosman et al., offers an additional path to address burnout and well-being in pediatricians through increasing of hours in more satisfying professional activities. While “satisfaction” was the metric in this study, what lay at the root of that satisfaction may be deeper and more profound. What the study does not measure is that the less-burned out physicians who felt greater satisfaction may have also felt a greater sense of meaning in their lives.

Grossman et al. rightly urge health care managers to encourage diversification of the pediatrician’s job by enabling greater engagement in the identified ‘anti- burnout’ professional activities, however more can and should be done. Physicians themselves should take an active role in both the seeking of, and connection to, meaning. Burnout and frustration, understandably, may have led some doctors to possess a sense of cynicism that has obscured meaning in their lives. If physicians cannot find a path to meaning on their own, they should seek colleague partners, coaches, or therapists to assist. Physicians can advocate for programs to reduce work-force stressors, but they can also advocate for formal programs such as Healers Arts programs, Schwartz rounds, and narrative medicine programs to help reconnect to meaning in their daily clinical work. Brief courses in cognitive behavioral techniques may also help in combating problematic mindsets endemic in medicine such as negativity bias, maladaptive perfectionism, and pessimistic explanatory style. With effort, a growth mindset, and when needed, guidance and some reinforcement, these negative and toxic mindsets can diminish; they can fade, and further open physicians to the healing power of meaning.

Physician burnout and depression have been recognized as serious international problems. The secondary costs of poor physician mental health are substantial, with evidence of links to increased medical errors, decreased patient satisfaction, reduced productivity and job satisfaction, and higher physician turnover [1–6]. Interventions to address this problem are being implemented across the globe, and they can be split into two broad categories: those that focus on the individual, and those that address the work environment. Individual-focused programs have been more at the forefront of wellness programs that often include instruction in mindfulness, meditation, yoga, nutrition classes, and exercise delivered in-person and online [7, 8]. Efforts in the work environment have, to a great a degree, focused on stressors such as administrative and documentary burden, electronic health records, and productivity pressures [5].

In their paper “Burnout and intentions to quit the practice among community pediatricians: Associations with specific professional activities”, Grossman et al. offer an additional path to address burnout and well-being in pediatricians that holds great potential [9]. The key finding in their study is illustrated in the following sentence: “The greater the discrepancy between the engagement of the pediatrician and the satisfaction felt in the measured professional activities, the greater was the burnout level (*p* <0.01)”.

While “satisfaction” was the metric in this study, what lay at the root of that satisfaction may be deeper and more profound. What the study does not measure—and what remains a plausible theory-- is that the less-burned out physicians who felt greater satisfaction may have also felt a greater sense of meaning in their lives.

Viktor Frankl, the noted psychiatrist, neurologist, and author of *Man’s Search for Meaning.* believed that finding meaning in life could sustain one even in the most difficult circumstances. In the case of his book, this circumstance was the concentration camps [10].

“There is nothing in the world, I venture to say, that would so effectively help one to survive even the worst conditions as the knowledge that there is a meaning in one’s life. There is much wisdom in the words of Nietzsche: ‘He who has a *why* to live for can bear almost any *how*.’” (italics added).

Frankl believed that the same holds true outside the concentration camp—more broadly in life in general.

Dr. Grossman and his co-authors rightly urge health care managers to “encourage diversification of the pediatrician’s job by enabling greater engagement in the identified ‘anti- burnout’ professional activities, such as: participation in professional consultations, management, tutoring students and conducting research.” These are important steps to take, but more can be done. This issue—one that so deeply impacts physicians across the world-- should not be left just to scheduling of activities by managers. Physicians themselves can and should take an active role in both the seeking of, and connection to, meaning. This meaning may take the form of capital M Meaning- the sense that one’s life is making some difference in this world, but it also may be found in the little moments of meaning that are available to us in the micro-interactions we have with people every day. Burnout and frustration, understandably, may have led some physicians to possess a sense of cynicism that has obscured meaning in their lives. In some, this may have evolved into a sense of learned helplessness where they may ask themselves, why even try. But in a world where many may feel little autonomy in their professional lives, there is one final freedom that remains. Frankl has something to say about this as well in another profound quote [10].

“Everything can be taken from a man but one thing: the last of human freedoms - to choose one’s attitude in any given set of circumstances, to choose one’s own way.”

If physicians cannot imagine finding this path on their own (and many may not, given their stresses and their exhaustion), they should seek colleague partners in this work, or seek out therapists or coaches. Physicians can also be advocates for change at their own institutions. They can and should ask for programs to reduce work-force stressors, but they can also advocate for formal programs such as Healers Arts programs, Schwartz rounds, and narrative medicine programs to help collectively reconnect to feelings of meaning and purpose not just in areas such as research and teaching, but in daily clinical work in their interactions with their colleagues in health care and with their patients [11–14]. Brief courses in cognitive behavioral techniques may also help in combating problematic mindsets endemic in medicine such as negativity bias, maladaptive perfectionism, pessimistic explanatory style, and the cognitive distortions that fuel them. These mindsets and patterns of thinking are acquired over years, and are certainly understandable given the pressures and demands physicians face, but they may blind us to moments of beauty and of grace in life. With effort, a growth mindset, and perhaps a little guidance and some reinforcement, these negative and toxic mindsets can diminish; they can fade, and further open us to the healing power of meaning.

## Conclusions

Health care managers should work to increase physician engagement in satisfying professional activities, but physicians should also commit to trying to find greater satisfaction and meaning in their professional lives, Finally, institutions and health centers should commit resources, not just directly to promote well-being and satisfaction, but also to promote those elements that can serve as their foundatio: meaning, purpose, connection, and ultimately the sense of being a part of something bigger than oneself. Not a job, not a career, but a calling.

## References

[1] Shanafelt TD, Balch CM, Bechamps G (2010). Burnout and medical errors among American surgeons. Ann Surg.

[2] Haas JS, Cook EF, Puopolo AL, Burstin HR, Cleary PD, Brennan TA (2000). Is the professional satisfaction of general internists associated with patient satisfaction?. J Gen Intern Med.

[3] Dewa CS, Loong D, Bonato S, Thanh NX, Jacobs P (2014). How does burnout affect physician productivity? A systematic literature review. BMC Health Serv Res.

[4] Shanafelt TD, Balch CM, Bechamps GJ (2009). Burnout and career satisfaction among American surgeons. Ann Surg.

[5] West CP, Dyrbye LN, Shanafelt TD (2018). Physician burnout: contributors, consequences and solutions. J Intern Med.

[6] Dyrbye LN, Varkey P, Boone SL, Satele DV, Sloan JA, Shanafelt TD (2013). Physician satisfaction and burnout at different career stages. Mayo Clin Proc.

[7] Barnes N, Hattan P, Black DS, Schuman-Olivier Z (2017). An examination of mindfulness-based programs in US medical schools. Mindfulness..

[8] Shapiro SL, Astin JA, Bishop SR, Cordova M (2005). Mindfulness-based stress reduction for health care professionals: results from a randomized trial. Int J Stress Manage.

[9] Grossman Z, Chodick G, Kushnir T, Cohen HA, Chapnick G, Ashkenazi S (2019). Burnout and intentions to quit the practice among community pediatricians: associations with specific professional activities. Isr J Health Policy Res.

[10] Frankl V (2006). Man’s search for meaning. Boston. Mass: Beacon press.

[11] Rabow MW, Wrubel J, Remen RN (2007). Authentic community as an educational strategy for advancing professionalism: a national evaluation of the Healer’s art course. J Gen Intern Med.

[12] Lown BA, Manning CF (2010). The Schwartz center rounds: evaluation of an interdisciplinary approach to enhancing patient-centered communication, teamwork, and provider support. Acad Med.

[13] Charon R. Narrative medicine: honoring the stories of illness: Oxford University Press; 2008 Feb 14.

[14] Charon R (2001). Narrative medicine: a model for empathy, reflection, profession, and trust. Jama..

